# Engaging the private sector to improve antimicrobial use in the community: experience from accredited drug dispensing outlets in Tanzania

**DOI:** 10.1186/2052-3211-7-11

**Published:** 2014-09-17

**Authors:** Richard Valimba, Jafary Liana, Mohan P Joshi, Edmund Rutta, Martha Embrey, Maganga Bundala, Bryceson Kibassa

**Affiliations:** Management Sciences for Health, Dar es Salaam, Tanzania; Management Sciences for Health/Systems for Improved Access to Pharmaceuticals and Services (SIAPS) Program, Arlington, VA USA; Management Sciences for Health, Arlington, VA USA; Tanzania Food and Drugs Authority, Dar es Salaam, Tanzania

**Keywords:** Antimicrobial resistance, Drug seller, Medicine use, Private sector, Tanzania

## Abstract

**Objectives:**

A public-private partnership in Tanzania launched the accredited drug dispensing outlet (ADDO) program to improve access to quality medicines and pharmaceutical services in rural areas. ADDO dispensers play a potentially important role in promoting the rational use of antimicrobials, which helps control antimicrobial resistance (AMR). The study objectives were to 1) improve dispensing practices of antimicrobials, 2) build ADDO dispensers’ awareness of the consequences of misusing antimicrobials, and 3) educate consumers on the correct use of antimicrobials through the use of printed materials and counseling.

**Methods:**

Our intervention targeted ADDO dispensers and community members in Kilosa district. We promoted AMR awareness using posters hung in public places, health facilities, and ADDOs; sensitizing 84 health care providers on AMR issues; and providing training and on-site support for 124 ADDO dispensers to increase their AMR knowledge and dispensing skills. Baseline and endline assessments included direct observation of dispensers’ practices; interviews with ADDO dispensers (71 at baseline and 68 at endline) regarding dispensing experiences; 230 exit interviews with ADDO customers regarding use of antimicrobials during monitoring visits; and review of ADDO records. Indicators were based on product availability, dispensing practices, customers’ knowledge of how to take their medicines, and dispenser and public awareness of the AMR threat.

**Results:**

Availability of tracer antimicrobials increased by 26% (p = 0.0088), and the proportion of ADDOs with unauthorized items decreased from 53% to 13% (p = 0.0001). The percentage of ADDO dispensers following good dispensing practices increased from an average of 67% in the first monitoring visit to an average of 91% during the last visit (p = 0.0001). After the intervention, more dispensers could name more factors contributing to AMR and negative consequences of inappropriate antimicrobial use, and over 95% of ADDO customers knew important information about the medicines they were dispensed.

**Conclusions:**

Providing educational materials and equipping ADDO dispensers with knowledge and tools helps significantly improve community medicine use and possibly reduces AMR. The number of community members who learned about AMR from ADDO dispensers indicates that they are an important source of information on medicine use.

## Introduction

Many people in sub-Saharan Africa seek health care from the private sector for reasons such as convenience and better availability of medicines [1]. People who use drug shops and pharmacies come in requesting specific medicines for self-treatment or advice from the drug seller on treatment or with a prescription to fill from a health care provider [2–4]. Therefore, retail drug sellers may be first people’s contact regarding health care for themselves or their children if that is their primary medicine source [5].

Historically in Tanzania, the local retail drug shop called *duka la dawa baridi* was authorized by the Tanzania Pharmacy Board to provide nonprescription medicines in the private sector. However, a 2001 assessment showed that many shops sold prescription drugs illegally and that drug sellers were generally unqualified and untrained [6]. In response, the Strategies for Enhancing Access to Medicines Program, funded by the Bill & Melinda Gates Foundation, collaborated with the Tanzania Food and Drugs Authority (TFDA) to develop and launch the accredited drug dispensing outlet (ADDO) program in 2003. The program’s goal was to improve access to affordable, quality essential medicines and pharmaceutical services in the *duka la dawa baridi*, which were mainly located in areas where few or no registered pharmacies exist. To achieve this goal, the program took a holistic approach that combined training, accreditation, business incentives, and regulatory enforcement with efforts to increase consumer demand for quality products and services. A key incentive for owners to become accredited was to legally allow ADDO dispensers to sell a limited list of essential prescription-only antimicrobials [7].

Successful results of the pilot in Tanzania’s Ruvuma region provided proof that ADDOs could improve access to quality medicines and pharmaceutical services [8]. Based on program evaluations, the Ministry of Health and Social Welfare approved a plan to roll out the ADDO concept in mainland Tanzania. As the program has taken off, many have recognized the potential of ADDOs not only to increase access to essential medicines, but also to serve as a platform for community-based public health interventions, such as distributing subsidized antimalarials [9]. As a result, numerous organizations and programs have played a role in expanding both the services that ADDOs provide and their geographic reach—over 6,000 ADDOs currently serve all mainland regions in Tanzania [10].

As a major source of essential medicines and health services in the community, ADDO dispensers play a potentially important role in promoting rational use of antimicrobials, which helps control antimicrobial resistance (AMR). Poor antimicrobial use includes overuse, prescription of wrong antimicrobials, and not taking a full dose of the dispensed medicines [11, 12]. As part of an initiative to support advocacy and containment of AMR in the community, the U.S. Agency for International Development-funded Strengthening Pharmaceutical Systems Program focused on ADDOs. Our objectives were to improve ADDO dispensers’ antimicrobial dispensing and counseling practices by providing training, job aids, educational materials, and regular supervision and to increase awareness of AMR in the surrounding community.

## Methods

### Study sites

Our intervention targeted ADDO dispensers and community members in Kilosa district, which is in the western part of the Morogoro region with a population of 438,175 people [13]. Kilosa has 50 public health facilities, 18 private clinics, and 125 ADDOs. We promoted AMR awareness using posters hung in public places, health facilities, and ADDOs; sensitizing 84 health care providers on AMR issues; and providing training and on-site support for 124 ADDO dispensers to increase their AMR knowledge and dispensing skills.

### Data collection and analysis

We performed baseline and endline assessments before and after the AMR intervention. In addition, as part of the on-site supervisory monitoring strategy, we collected data using a combination of review of dispensing records, in-person interviews with dispensers and ADDO customers, and observation of customer encounters. The data collected was used to help track progress during the intervention based on six indicators to measure the ADDO dispensers’ ability to:

1. Correctly interpret a prescription brought in by the client
2. Appropriately prepare needed medicines to dispense
3. Provide correct instructions to clients on how to use the dispensed medicines
4. Provide information on the medicine’s possible side effects
5. Provide other information if appropriate
6. Double-check the clients’ understanding of the information they received regarding correct medicine use

We used the same data collection instrument for the baseline and endline assessments, and a different instrument during the three monitoring visits. Pharmacy students collected baseline and endline data, and pharmacist or medical professionals conducted the three supervisory visits. A list of 14 tracer antimicrobials was used to assess availability of antimicrobials that ADDOs are legally allowed to stock. Table 1 summarizes the data gathering activities.

**Table 1:** **Data collection for AMR intervention in Kilosa district**

Source	Baseline assessment	Monitoring visit 1 (N = 81)	Monitoring visit 2 (N = 93)	Monitoring visit 3 (N = 88)	Endline assessment
Number of ADDO dispensers interviewed	71	41	42	47	68
Number of ADDO customers interviewed		80	92	58	
Number of ADDO customer interactions observed		40	48	52	
Number of ADDO dispensing records reviewed for diarrhea		251	295	258	
Number of ADDO dispensing records reviewed for upper respiratory infection		365	498	447	

We analyzed the data using Excel. Prior to collecting data, we sought approval from the TFDA and Pharmacy Council. After being briefed on the purpose of the study, ADDO dispensers were asked to participate and upon consent were interviewed. Data collectors verbally assured participants of the confidentiality of information collected, their anonymity, and the freedom to withdraw at any time during the process. Additionally, prior to data collection, we held meetings with district officials to brief them about the study.

### Baseline assessment

In August 2008, we did a baseline assessment of ADDO dispensing practices, dispensers’ knowledge of factors that contribute to the development of AMR and consequences of inappropriate use of antimicrobials. Using a structured questionnaire, we interviewed 71 ADDO dispensers from randomly chosen shops in the district. In addition, we reviewed ADDO dispensing records and observed dispensing practices. We used the information gathered during the baseline survey to design the intervention. An endline assessment in October 2010 determined if the intervention had changed behavior of ADDO dispensers and improved appropriate use of antimicrobials.

### Job aids and educational materials

We developed job aids including an antimicrobial dispensing guide, counter-top cards with information for customers on one side and information for ADDO dispensers on the other, and rubber stamps to label medicine packages and help dispensers provide counseling on appropriate use of antimicrobials at home. The information on the stamp included the patient’s name, the medicine name, strength, and quantity dispensed, how to take the medicine, the date dispensed, and the ADDO name. A poster featured information in Kiswahili to increase awareness of AMR and appropriate medicine use among the public. The materials were pretested with both dispensers and community members. Two thousand public information posters were displayed in all ADDOs, all the district’s health facilities, and public gathering places, and 300 counter-top cards, 300 dispensing guides, lists of prescription medicines ADDOs are authorized to dispense, and rubber stamps were distributed to all the ADDOs in the district.

### Sensitization seminars

We collaborated with TFDA and the Kilosa district pharmacist to launch and distribute the job aids and educational materials to 124 ADDO dispensers (99% of total), prescribers from all 84 health facilities in Kilosa, and 8 members of the district’s Council Health Management Team, who are responsible for supervising health facilities and ADDOs. During the December 2009 seminar, we shared the AMR baseline assessment results and presented the new materials for participants to discuss. At the end of the seminar, participants received printed materials to take to their work places. District health staff also received a supervision checklist to help support ADDO dispensers on issues related to AMR.

### Follow-up monitoring visits

We conducted 3 visits in the 10 months after the intervention launch to provide on-site support to ADDO dispensers, to collect monitoring information from ADDOs, to interview ADDO customers, and to continue to distribute materials to health facilities and other public gathering places in the district. The visits to 81, 93, and 88 individual ADDOs were conducted in March, June, and October 2010, respectively; at those times, we provided on-site supervision to 93, 101, and 110 dispensers. During the visits, ADDO dispensers were reminded to follow good dispensing practices such as—

- Reviewing customer’s prescription
- Writing the correct information on medication labels
- Packaging medications from containers using clean equipment and not hands
- Providing accurate instructions on medicine use to customers
- Checking customers’ understanding by asking for feedback

To assess whether ADDO dispensers used these good dispensing practices, we observed interactions with customers or checked dispensers’ knowledge using a structured checklist, which had been pretested. We also carried out exit interviews with 80, 92, and 58 ADDO customers after each monitoring visit to measure their awareness regarding AMR and to learn about their experiences with the dispenser.

### Endline assessment

In October 2010, 10 months after the start of the intervention, we conducted an endline evaluation to determine the intervention’s results, to highlight challenges encountered during the implementation, and to provide recommendations that would help guide future intervention scale-up plans. The survey comprised assessment data from 65 ADDOs, including interviews with 68 dispensers, using a structured questionnaire to assess dispensers’ knowledge about AMR and its contributing factors and knowledge about the negative consequences of inappropriate antimicrobial use. We included all of the same 71 randomly selected ADDOs at the baseline assessment, but six shops were closed.

### Dissemination workshop

After the endline data analysis, we organized a one-day dissemination workshop with 50 key stakeholders to share findings from the AMR activities. Stakeholders included ADDO dispensers and owners, health facility staff, and Kilosa district community members. Participants also included representatives from TFDA, National Malaria Control Program, Pharmacy Council, Ministry of Health and Social Welfare, other implementing partners, and the Muhimbili University School of Pharmacy. In addition to sharing the evaluation results, we used the meeting as a forum to discuss options and recommendations regarding a strategy for possible scale-up.

## Results

To measure success or failure of the ADDO AMR initiative, throughout the implementation period we monitored changes based on the six indicators mentioned above. Indicators addressed product availability, dispensing practices, and dispenser and customer and public knowledge of AMR issues.

### Availability of antimicrobials

Monitoring results showed an increase in availability of these antimicrobials from an average of 62% at the first monitoring visit to an average of 78% at the last monitoring visit (p = 0.0088). In addition, during monitoring visits, dispensers were reminded to stop stocking antimicrobials that are not allowed. As a result, availability of unauthorized medicines decreased significantly (p = 0.0001) (Table 2).

**Table 2:** **Percentage of ADDOs stocking antimicrobials not on the ADDO approved medicine list**

Number of unauthorized products available	Monitoring visit 1 (%)	Monitoring visit 2 (%)	Monitoring visit 3 (%)
	(N = 81)	(N = 93)	(N = 88)
0	47	79	87*
1–3	38	14	13*
4–6	13	5	0*
7–9	2	2	0

*Statistically significant (p < 0.05).

### Dispensing practices

After the intervention, results showed that the percentage of ADDO dispensers using good dispensing practices increased from an average of 67% in the first follow-up visit to an average of 91% during the last follow-up visit (p = 0.0001). That means almost all dispensers met all six indicators of good dispensing practices as recommended in the dispensing guide, thereby ensuring that customers received the correct instructions for using medicines.

### Correct labeling

A correct medicine label helps remind a customer of how to take the dispensed antimicrobials appropriately. During the baseline assessment, 18% of ADDO dispensers were using rubber stamps for labeling. However, the stamps were not uniform, and some lacked important information. Consequently, we distributed standardized rubber stamps for medicine labeling during the first follow-up visit and then assessed dispensers’ appropriate use of the stamps in the second and third follow-up visits. When we interviewed customers exiting ADDOs, we checked to see whether their medicine labels included the name of the medicine, strength, and instructions for use. At the second monitoring visit, 95% of the packages included the name of the medicine; 77% included the strength, and 93% included instructions for use; at the third visit, the proportions were 98%, 91%, and 98%, respectively.

### Counseling information

The intervention focused on educating dispensers on the importance of providing adequate instructions for medicine use to customers and how these instructions and medicine use influence AMR. At endline, the majority of dispensers who were interviewed (an average of 89%) were aware of the important information to be covered when dispensing medicines to customers compared to 58% of dispensers during the baseline assessment (p = 0.0002) (Table 3).

**Table 3:** **Percentage of ADDO dispensers who knew what medication information to discuss with customers**

Topic discussed during medicine counseling	Baseline (%) (N = 71)	Endline (%) (N = 68)
Name of medicine	38	88*
What condition medicine treats	15	85*
How to take medicine	96	100
When to take medicine	94	98
Proper dose	73	95*
Proper storage	55	92*
Importance of completing course	55	100*
Not drinking alcohol (if applicable)	49	71*
Possible side effects	47	77*

*p < 0.05.

### Customers’ knowledge of how to take dispensed medicines

We then collected information on ADDO customers’ knowledge of how to properly take their medicines including dose (quantity), dosage (number per day), and course (duration) of the medicine that they had been dispensed. The results from data collected from ADDO customers during monitoring visits showed that ADDO dispensers improved the quality of their counseling, so that customers were able to understand how to properly take the antimicrobials they had been dispensed (Table 4).

**Table 4:** **Percentage of ADDO customers who knew how to properly take their medicines**

	Monitoring visit 1 (%) (N = 80)	Monitoring visit 2 (%) (N = 92)	Monitoring visit 3 (%) (N = 58)
Percentage of customers who knew drug dose (how many)	93	96	98
Percentage of customers who knew drug dosage (how often)	71	88	95*
Percentage of customers who knew treatment course (how many days)	68	83	100*

*p < 0.05.

### Appropriate treatment of common health conditions

**Figure 1:**
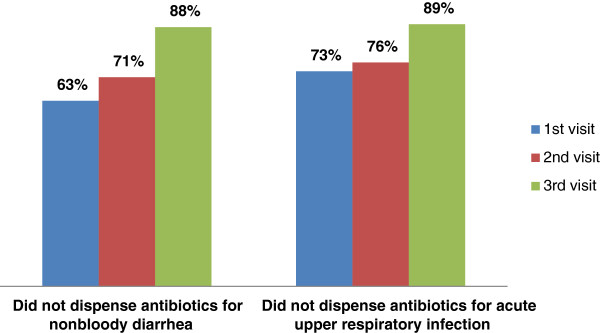
**Percentage of ADDO dispensers who appropriately did not dispense antibiotics for common conditions.**

Although ADDOs are allowed to sell and dispense select antibiotics used to treat common conditions, dispenser training emphasizes appropriate use, including not recommending antimicrobials to treat conditions such as acute upper respiratory infections and uncomplicated nonbloody diarrhea, which do not require antibiotics. Results from reviews of dispenser-managed treatment records during the three follow-up monitoring visits showed statistically significant improvements; for example, dispensing antibiotics for nonbloody diarrhea decreased from 37% at the first visit to 12% at the last visit (p = 0.0002), as indicated in Figure 1. Similar decreases in inappropriate treatment of acute upper respiratory infections occurred over the monitoring period (p = 0.0088).

### Dispensers’ awareness of consequences of inappropriate antimicrobial use and factors contributing to AMR

**Figure 2:**
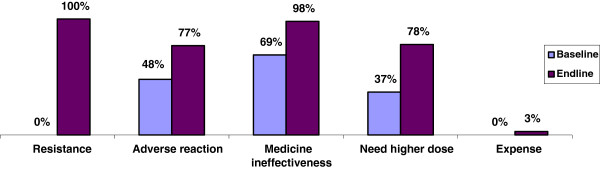
**Percentage of ADDO dispensers listing negative consequences of inappropriate antimicrobial use.**

Overall, at endline, the dispensers included more negative effects in their lists of the consequences for inappropriate medicine use (five compared with three at baseline), and a higher percentage of dispensers mentioned each consequence (Figure 2).

In addition, ADDO dispensers’ awareness of factors that contribute to AMR increased as a result of the sensitization and follow-up visits. After the intervention, a higher percentage of dispensers could name more AMR factors (Table 5).

**Table 5:** **Percentage of dispensers listing factors contributing to AMR**

Factors contributing to AMR	Baseline (%)	Endline (%)
	(N = 71)	(N = 68)
Incomplete course	83	100*
Insufficient amount of medicine	66	89*
Poor quality of medicine	49	74*
Wrong medicine	44	91*
Nonadherence	7	3
Take someone else’s medicine	6	94*

*p < 0.05.

## Discussion

The ADDO program is an innovative public-private partnership that increases access to quality pharmaceuticals and services—especially in rural and periurban locations. This project demonstrated the feasibility of using the ADDO platform to translate the World Health Organization’s strategy to control AMR into practice at the community level and with a modest use of resources [14]. Although carrying out such interventions in the private sector is challenging [15–17], the ADDO platform facilitated this activity. For example, we were able to use the TFDA’s supervisory structure to implement the intervention, and we used the existing ADDO registers to assess the appropriateness of treatment over time and triangulate that data with an assessment of dispenser knowledge and other data collection.

Overall, the availability of authorized antimicrobials increased, while availability of unauthorized antimicrobials decreased. The availability of the unauthorized medicines may contribute to inappropriate use because dispensers have not been trained on how to dispense them. Although it is possible that dispensers hid those medicines from the supervisors, knowing that they were not allowed, we assume that would have occurred during all the visits uniformly, rather than showing the steady decrease that was observed.

At baseline, few ADDOs had rubber stamps to help them label medicine packages appropriately. Although using a standardized stamp is not required, clear and comprehensive labeling is a requirement, and the stamp was very effective at improving the quality of medicine labels, with almost 100% including the important information on the labels.

In terms of the quality of dispensing, many more dispensers knew by endline which instructions they should give customers and how these instructions and how customers’ inappropriate use of medicines can contribute to AMR; for example, whereas none of the dispensers mentioned AMR as a consequence of poor antimicrobial use at baseline, they all mentioned it at endline. Our interviews with customers exiting the shops also showed that the dispensers had explained how to take dispensed medicines correctly. By the third visit, almost all (over 95%) of customers interviewed could correctly report the dose, dosage, and duration of treatment of the medicines they received from ADDOs. In addition, significantly fewer customers were being dispensed unneeded antimicrobials for simple diarrhea and acute respiratory infections—decreasing their out-of-pocket expenditures as well as reflecting appropriate practices.

In the workshop to present the study results, stakeholders recommended that the AMR job aids developed for counseling and labeling should be made mandatory tools in ADDOs and that the AMR concept should be incorporated into the ongoing ADDO training curriculum or into continuing education for dispensers. However, despite the encouraging results achieved by this AMR pilot, ADDO customers still ask for and clinicians prescribe antimicrobials for common illnesses such as diarrhea and upper respiratory tract infections. Because containing AMR requires a multifaceted effort, strengthening collaboration and partnerships with different stakeholders will help leverage resources for consumer awareness activities.

## Conclusions

Critics of private sector health care have argued that providers are motivated more by profit than by public health; however, we found that ADDO dispensers are anxious to have the correct knowledge regarding medicine use and are proud to be seen as a community resource on health. Providing educational materials and messages and equipping drug dispensers with knowledge and tools help improve community medicine use and possibly help reduce AMR. Using dispensers and health facility staff to distribute AMR educational materials promoted community awareness about the existence and consequences of the AMR threat and how improper self-medication can contribute to the threat.
